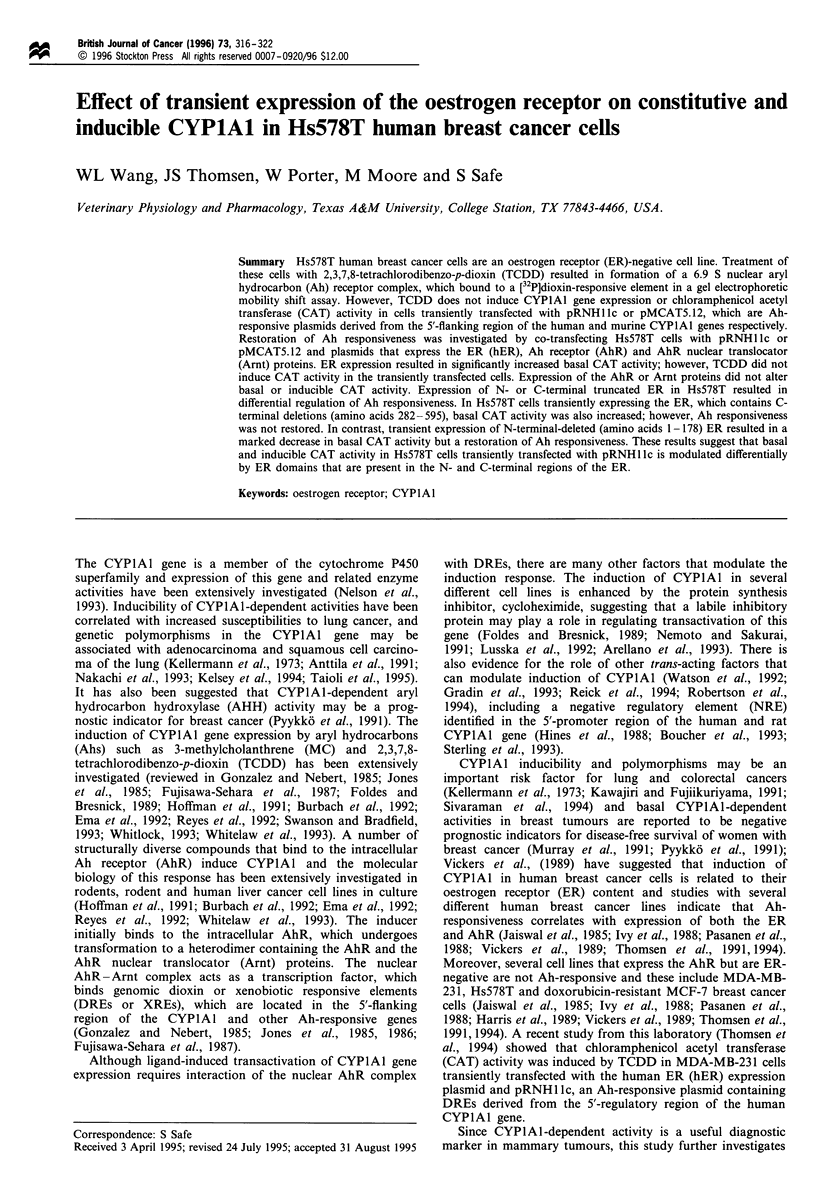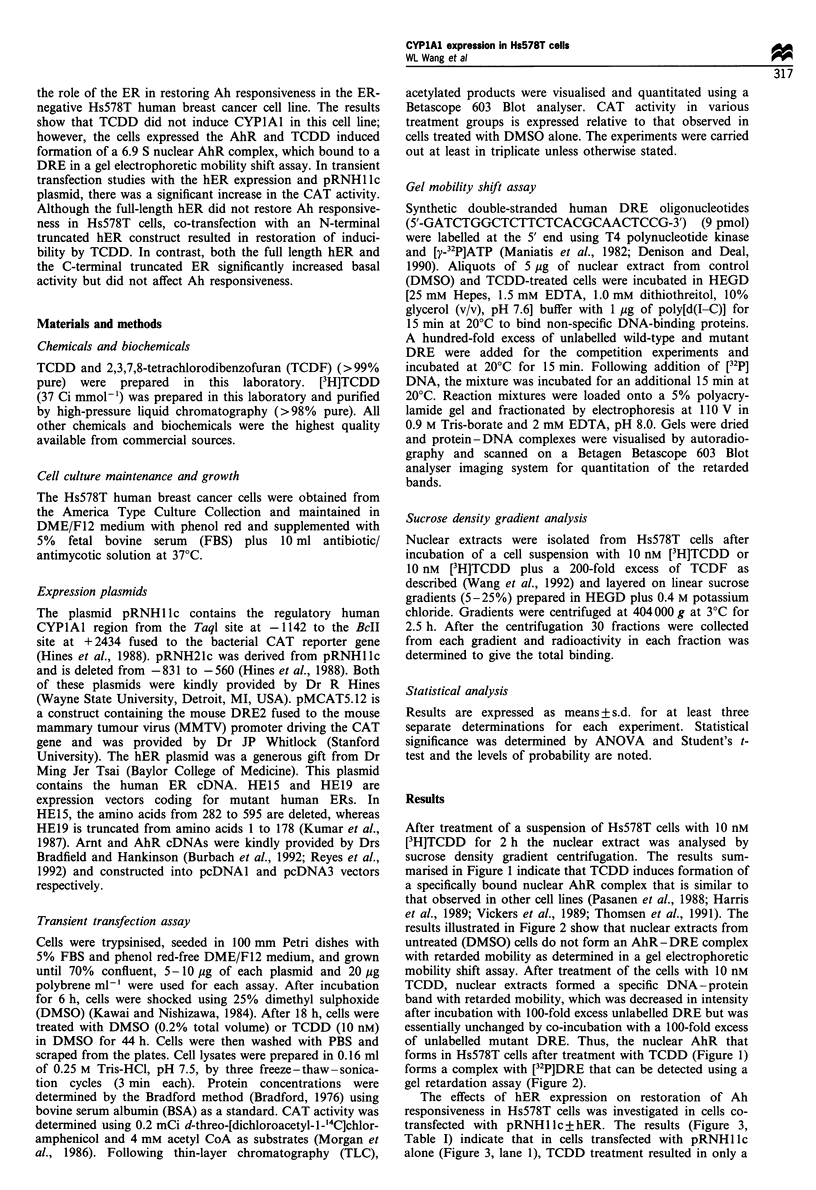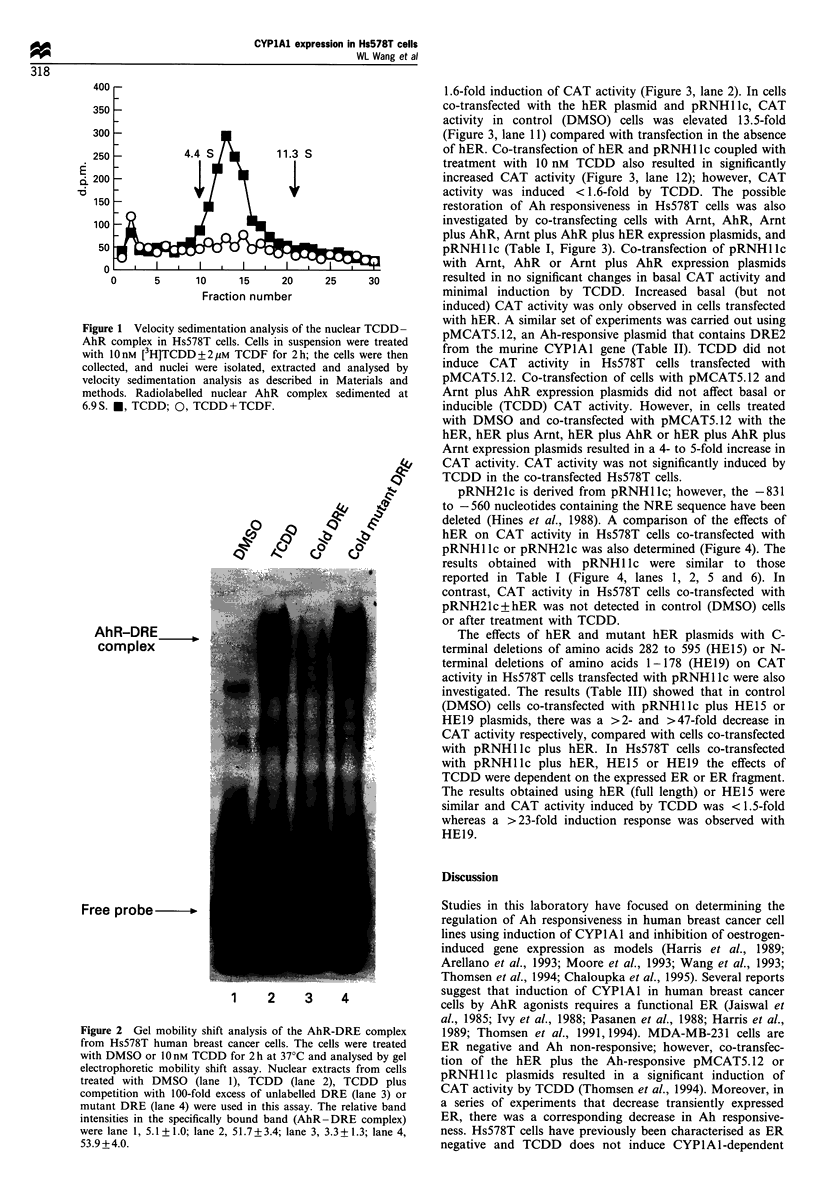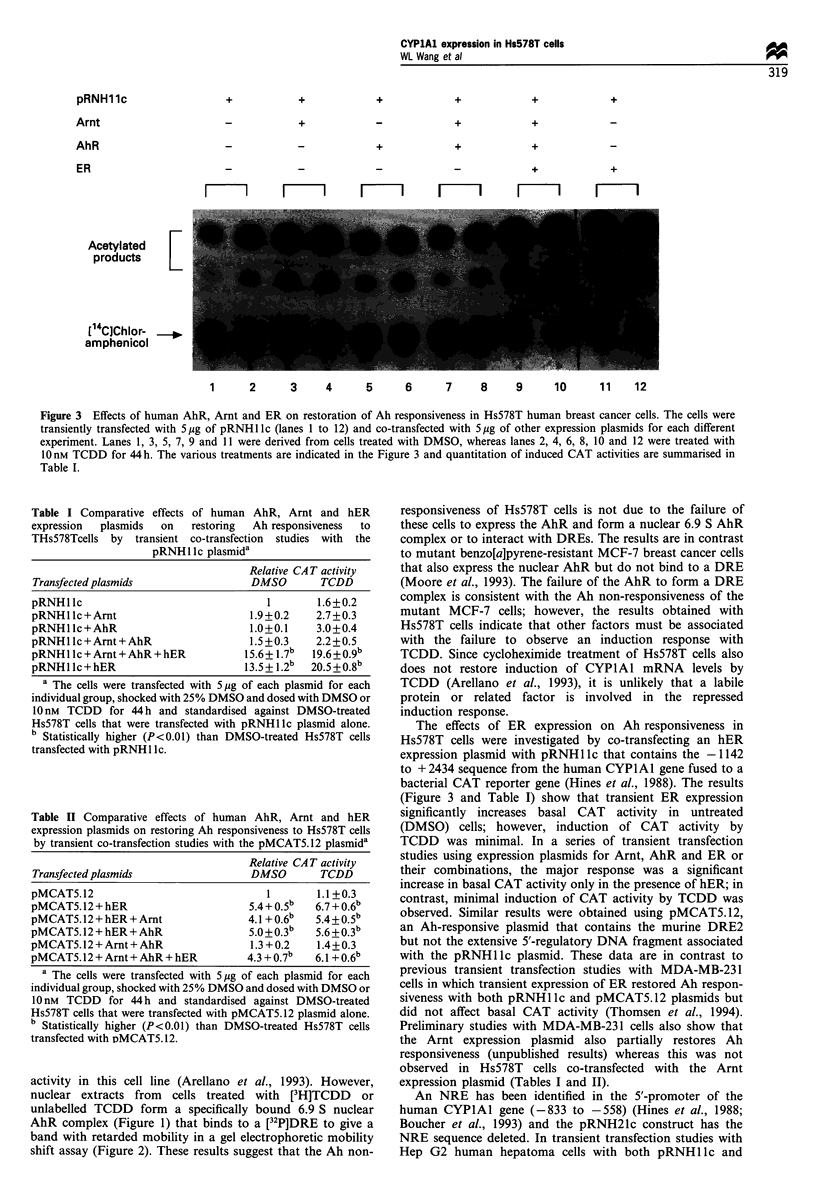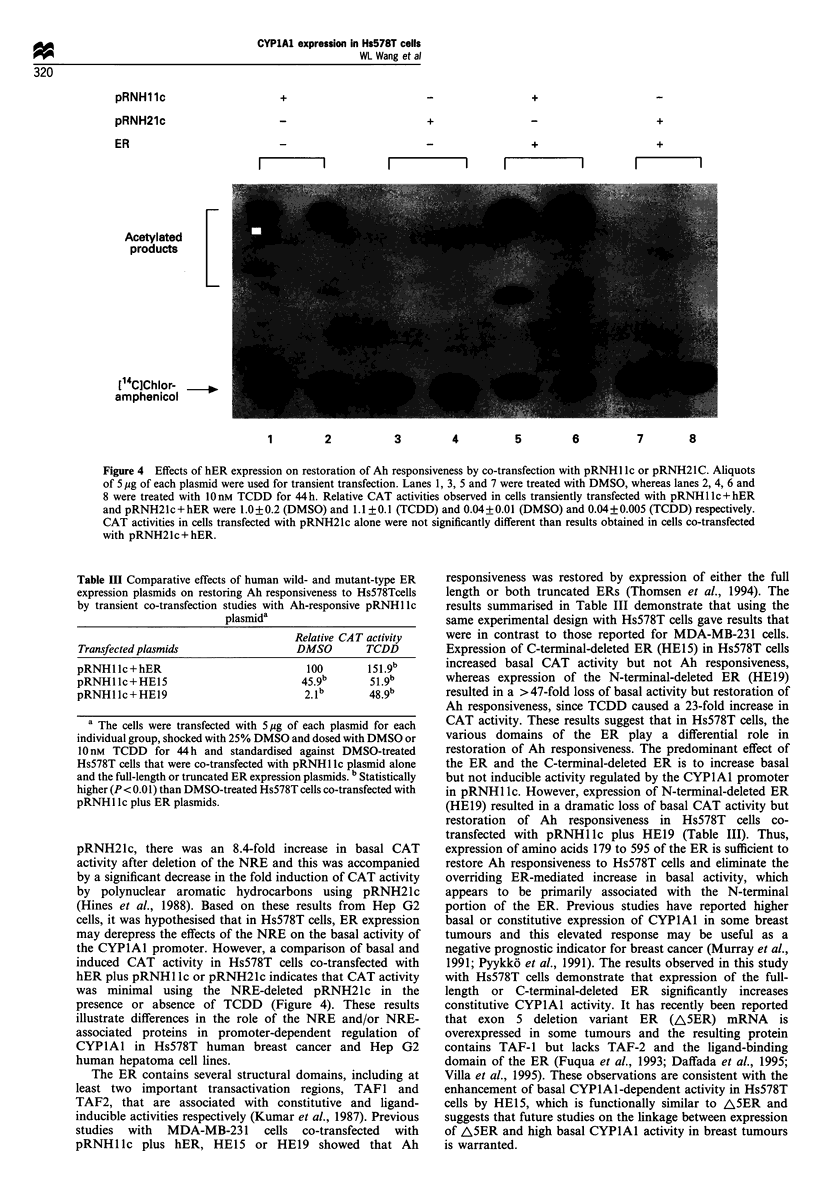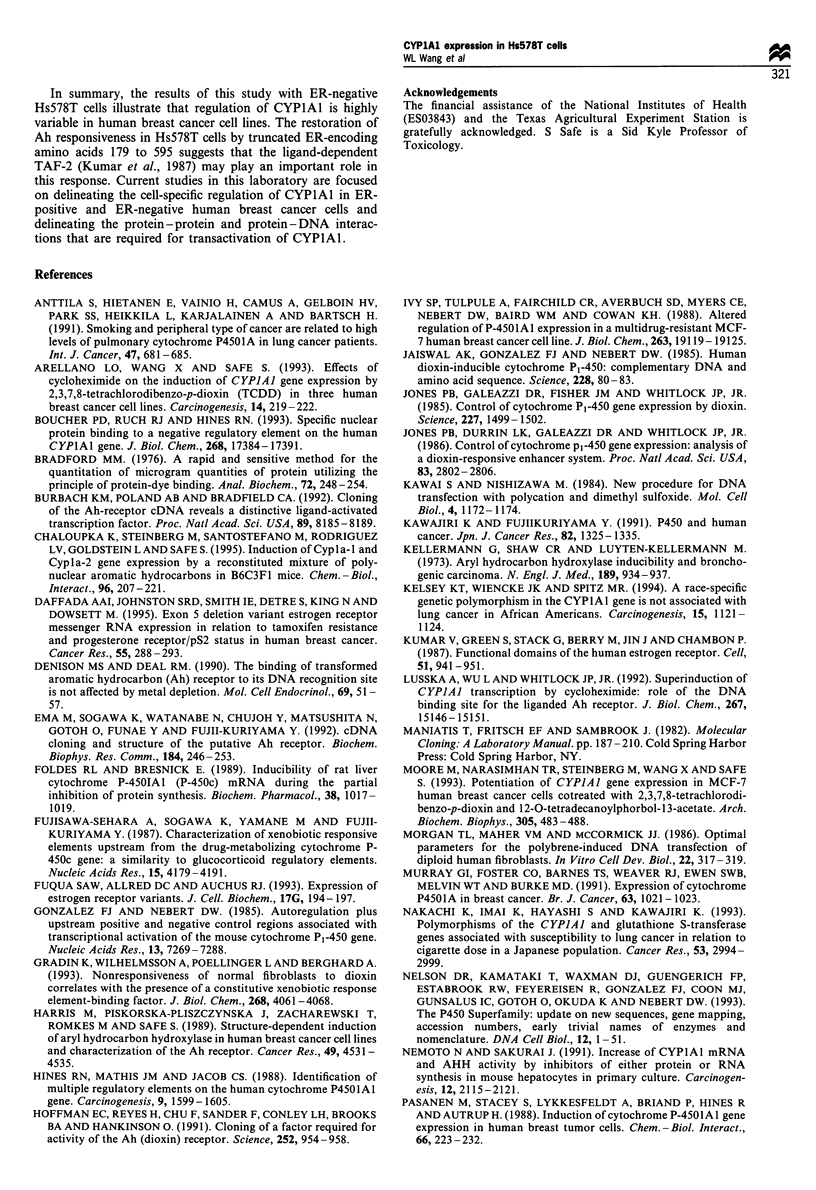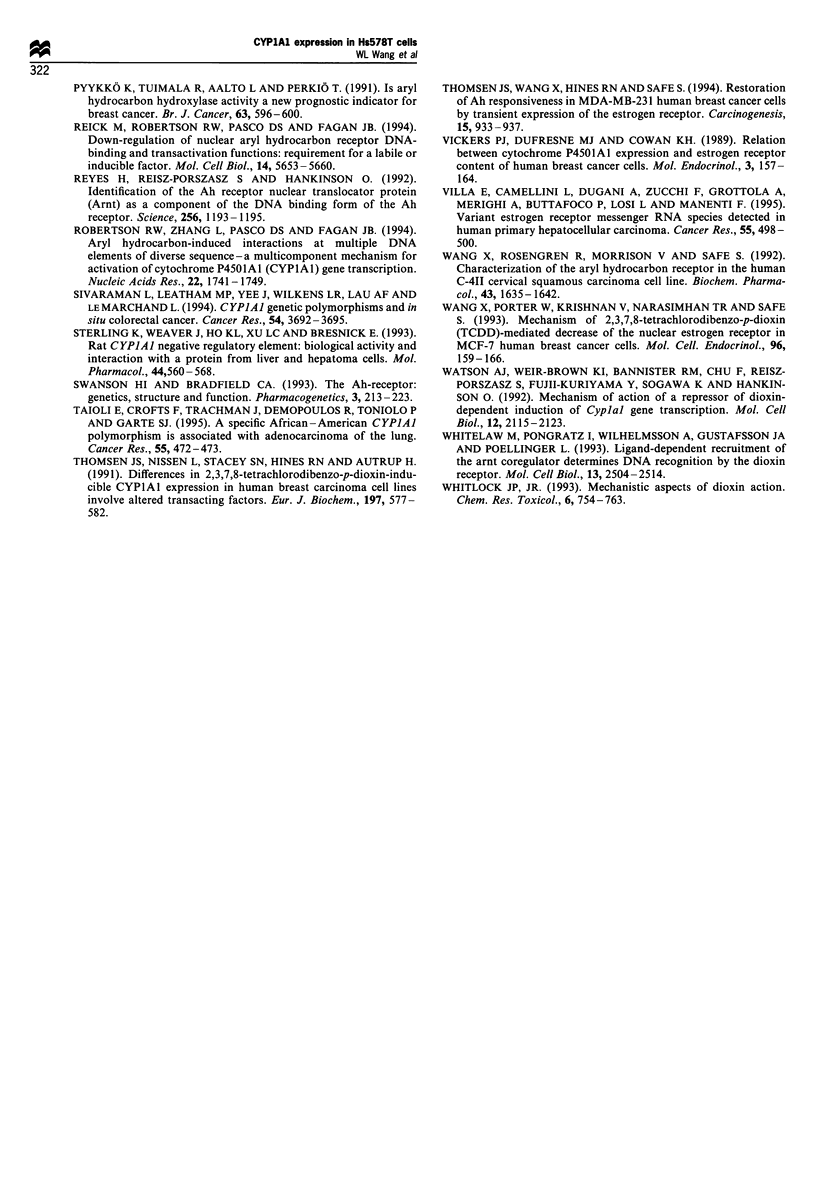# Effect of transient expression of the oestrogen receptor on constitutive and inducible CYP1A1 in Hs578T human breast cancer cells.

**DOI:** 10.1038/bjc.1996.55

**Published:** 1996-02

**Authors:** W. L. Wang, J. S. Thomsen, W. Porter, M. Moore, S. Safe

**Affiliations:** Veterinary Physiology and Pharmacology, Texas A&M University, College Station 77843-4466, USA.

## Abstract

**Images:**


British Journal of Cancer (1996) 73, 316-322
© 1996 Stockton Press All rights reserved 0007-0920/96 $12.00

### Effect of transient expression of the oestrogen receptor on constitutive and inducible CYP1A1 in Hs578T human breast cancer cells

WL Wang, JS Thomsen, W Porter, M Moore and S Safe

*Veterinary Physiology and Pharmacology, Texas A&M University, College Station, TX 77843-4466, USA.*

**Summary** Hs578T human breast cancer cells are an oestrogen receptor (ER)-negative cell line. Treatment of these cells with 2,3,7,8-tetrachlorodibenzo-*p*-dioxin (TCDD) resulted in formation of a 6.9 S nuclear aryl hydrocarbon (Ah) receptor complex, which bound to a [$^{32}$P]dioxin-responsive element in a gel electrophoretic mobility shift assay. However, TCDD does not induce CYP1A1 gene expression or chloramphenicol acetyl transferase (CAT) activity in cells transiently transfected with pRNH11c or pMCAT5.12, which are Ah-responsive plasmids derived from the 5'-flanking region of the human and murine CYP1A1 genes respectively. Restoration of Ah responsiveness was investigated by co-transfecting Hs578T cells with pRNH11c or pMCAT5.12 and plasmids that express the ER (hER), Ah receptor (AhR) and AhR nuclear translocator (Arnt) proteins. ER expression resulted in significantly increased basal CAT activity; however, TCDD did not induce CAT activity in the transiently transfected cells. Expression of the AhR or Arnt proteins did not alter basal or inducible CAT activity. Expression of N- or C-terminal truncated ER in Hs578T resulted in differential regulation of Ah responsiveness. In Hs578T cells transiently expressing the ER, which contains C-terminal deletions (amino acids 282-595), basal CAT activity was also increased; however, Ah responsiveness was not restored. In contrast, transient expression of N-terminal-deleted (amino acids 1-178) ER resulted in a marked decrease in basal CAT activity but a restoration of Ah responsiveness. These results suggest that basal and inducible CAT activity in Hs578T cells transiently transfected with pRNH11c is modulated differentially by ER domains that are present in the N- and C-terminal regions of the ER.

**Keywords:** oestrogen receptor; CYP1A1

The CYP1A1 gene is a member of the cytochrome P450 superfamily and expression of this gene and related enzyme activities have been extensively investigated (Nelson *et al.*, 1993). Inducibility of CYP1A1-dependent activities have been correlated with increased susceptibilities to lung cancer, and genetic polymorphisms in the CYP1A1 gene may be associated with adenocarcinoma and squamous cell carcinoma of the lung (Kellermann *et al.*, 1973; Anttila *et al.*, 1991; Nakachi *et al.*, 1993; Kelsey *et al.*, 1994; Taioli *et al.*, 1995). It has also been suggested that CYP1A1-dependent aryl hydrocarbon hydroxylase (AHH) activity may be a prognostic indicator for breast cancer (Pyykkö *et al.*, 1991). The induction of CYP1A1 gene expression by aryl hydrocarbons (Ahs) such as 3-methylcholanthrene (MC) and 2,3,7,8-tetrachlorodibenzo-*p*-dioxin (TCDD) has been extensively investigated (reviewed in Gonzalez and Nebert, 1985; Jones *et al.*, 1985; Fujisawa-Sehara *et al.*, 1987; Foldes and Bresnick, 1989; Hoffman *et al.*, 1991; Burbach *et al.*, 1992; Ema *et al.*, 1992; Reyes *et al.*, 1992; Swanson and Bradfield, 1993; Whitlock, 1993; Whitelaw *et al.*, 1993). A number of structurally diverse compounds that bind to the intracellular Ah receptor (AhR) induce CYP1A1 and the molecular biology of this response has been extensively investigated in rodents, rodent and human liver cancer cell lines in culture (Hoffman *et al.*, 1991; Burbach *et al.*, 1992; Ema *et al.*, 1992; Reyes *et al.*, 1992; Whitelaw *et al.*, 1993). The inducer initially binds to the intracellular AhR, which undergoes transformation to a heterodimer containing the AhR and the AhR nuclear translocator (Arnt) proteins. The nuclear AhR-Arnt complex acts as a transcription factor, which binds genomic dioxin or xenobiotic responsive elements (DREs or XREs), which are located in the 5'-flanking region of the CYP1A1 and other Ah-responsive genes (Gonzalez and Nebert, 1985; Jones *et al.*, 1985, 1986; Fujisawa-Sehara *et al.*, 1987).

Although ligand-induced transactivation of CYP1A1 gene expression requires interaction of the nuclear AhR complex with DREs, there are many other factors that modulate the induction response. The induction of CYP1A1 in several different cell lines is enhanced by the protein synthesis inhibitor, cycloheximide, suggesting that a labile inhibitory protein may play a role in regulating transactivation of this gene (Foldes and Bresnick, 1989; Nemoto and Sakurai, 1991; Lusska *et al.*, 1992; Arellano *et al.*, 1993). There is also evidence for the role of other *trans*-acting factors that can modulate induction of CYP1A1 (Watson *et al.*, 1992; Gradin *et al.*, 1993; Reick *et al.*, 1994; Robertson *et al.*, 1994), including a negative regulatory element (NRE) identified in the 5'-promoter region of the human and rat CYP1A1 gene (Hines *et al.*, 1988; Boucher *et al.*, 1993; Sterling *et al.*, 1993).

CYP1A1 inducibility and polymorphisms may be an important risk factor for lung and colorectal cancers (Kellermann *et al.*, 1973; Kawajiri and Fujiikuriyama, 1991; Sivaraman *et al.*, 1994) and basal CYP1A1-dependent activities in breast tumours are reported to be negative prognostic indicators for disease-free survival of women with breast cancer (Murray *et al.*, 1991; Pyykkö *et al.*, 1991); Vickers *et al.*, (1989) have suggested that induction of CYP1A1 in human breast cancer cells is related to their oestrogen receptor (ER) content and studies with several different human breast cancer lines indicate that Ah-responsiveness correlates with expression of both the ER and AhR (Jaiswal *et al.*, 1985; Ivy *et al.*, 1988; Pasanen *et al.*, 1988; Vickers *et al.*, 1989; Thomsen *et al.*, 1991, 1994). Moreover, several cell lines that express the AhR but are ER-negative are not Ah-responsive and these include MDA-MB-231, Hs578T and doxorubicin-resistant MCF-7 breast cancer cells (Jaiswal *et al.*, 1985; Ivy *et al.*, 1988; Pasanen *et al.*, 1988; Harris *et al.*, 1989; Vickers *et al.*, 1989; Thomsen *et al.*, 1991, 1994). A recent study from this laboratory (Thomsen *et al.*, 1994) showed that chloramphenicol acetyl transferase (CAT) activity was induced by TCDD in MDA-MB-231 cells transiently transfected with the human ER (hER) expression plasmid and pRNH11c, an Ah-responsive plasmid containing DREs derived from the 5'-regulatory region of the human CYP1A1 gene.

Since CYP1A1-dependent activity is a useful diagnostic marker in mammary tumours, this study further investigates

Correspondence: S Safe
Received 3 April 1995; revised 24 July 1995; accepted 31 August 1995

the role of the ER in restoring Ah responsiveness in the ER-negative Hs578T human breast cancer cell line. The results show that TCDD did not induce CYP1A1 in this cell line; however, the cells expressed the AhR and TCDD induced formation of a 6.9 S nuclear AhR complex, which bound to a DRE in a gel electrophoretic mobility shift assay. In transient transfection studies with the hER expression and pRNH11c plasmid, there was a significant increase in the CAT activity. Although the full-length hER did not restore Ah responsiveness in Hs578T cells, co-transfection with an N-terminal truncated hER construct resulted in restoration of inducibility by TCDD. In contrast, both the full length hER and the C-terminal truncated ER significantly increased basal activity but did not affect Ah responsiveness.

### Materials and methods

### *Chemicals and biochemicals*

TCDD and 2,3,7,8-tetrachlorodibenzofuran (TCDF) (>99% pure) were prepared in this laboratory. [$^3$H]TCDD (37 Ci mmol$^{-1}$) was prepared in this laboratory and purified by high-pressure liquid chromatography (>98% pure). All other chemicals and biochemicals were the highest quality available from commercial sources.

### *Cell culture maintenance and growth*

The Hs578T human breast cancer cells were obtained from the America Type Culture Collection and maintained in DME/F12 medium with phenol red and supplemented with 5% fetal bovine serum (FBS) plus 10 ml antibiotic/antimycotic solution at 37°C.

### *Expression plasmids*

The plasmid pRNH11c contains the regulatory human CYP1A1 region from the *Taq*I site at −1142 to the *Bcl*I site at +2434 fused to the bacterial CAT reporter gene (Hines *et al*., 1988). pRNH21c was derived from pRNH11c and is deleted from −831 to −560 (Hines *et al*., 1988). Both of these plasmids were kindly provided by Dr R Hines (Wayne State University, Detroit, MI, USA). pMCAT5.12 is a construct containing the mouse DRE2 fused to the mouse mammary tumour virus (MMTV) promoter driving the CAT gene and was provided by Dr JP Whitlock (Stanford University). The hER plasmid was a generous gift from Dr Ming Jer Tsai (Baylor College of Medicine). This plasmid contains the human ER cDNA. HE15 and HE19 are expression vectors coding for mutant human ERs. In HE15, the amino acids from 282 to 595 are deleted, whereas HE19 is truncated from amino acids 1 to 178 (Kumar *et al*., 1987). Arnt and AhR cDNAs were kindly provided by Drs Bradfield and Hankinson (Burbach *et al*., 1992; Reyes *et al*., 1992) and constructed into pcDNA1 and pcDNA3 vectors respectively.

### *Transient transfection assay*

Cells were trypsinised, seeded in 100 mm Petri dishes with 5% FBS and phenol red-free DME/F12 medium, and grown until 70% confluent, 5–10 μg of each plasmid and 20 μg polybrene ml$^{-1}$ were used for each assay. After incubation for 6 h, cells were shocked using 25% dimethyl sulphoxide (DMSO) (Kawai and Nishizawa, 1984). After 18 h, cells were treated with DMSO (0.2% total volume) or TCDD (10 nM) in DMSO for 44 h. Cells were then washed with PBS and scraped from the plates. Cell lysates were prepared in 0.16 ml of 0.25 M Tris-HCl, pH 7.5, by three freeze–thaw–sonication cycles (3 min each). Protein concentrations were determined by the Bradford method (Bradford, 1976) using bovine serum albumin (BSA) as a standard. CAT activity was determined using 0.2 mCi *d*-threo-[dichloroacetyl-1-$^{14}$C]chloramphenicol and 4 mM acetyl CoA as substrates (Morgan *et al*., 1986). Following thin-layer chromatography (TLC), acetylated products were visualised and quantitated using a Betascope 603 Blot analyser. CAT activity in various treatment groups is expressed relative to that observed in cells treated with DMSO alone. The experiments were carried out at least in triplicate unless otherwise stated.

### *Gel mobility shift assay*

Synthetic double-stranded human DRE oligonucleotides (5′-GATCTGGCTCTTCTCACGCAACTCCG-3′) (9 pmol) were labelled at the 5′ end using T4 polynucleotide kinase and [γ-$^{32}$P]ATP (Maniatis *et al*., 1982; Denison and Deal, 1990). Aliquots of 5 μg of nuclear extract from control (DMSO) and TCDD-treated cells were incubated in HEGD [25 mM Hepes, 1.5 mM EDTA, 1.0 mM dithiothreitol, 10% glycerol (v/v), pH 7.6] buffer with 1 μg of poly[d(I–C)] for 15 min at 20°C to bind non-specific DNA-binding proteins. A hundred-fold excess of unlabelled wild-type and mutant DRE were added for the competition experiments and incubated at 20°C for 15 min. Following addition of [$^{32}$P] DNA, the mixture was incubated for an additional 15 min at 20°C. Reaction mixtures were loaded onto a 5% polyacrylamide gel and fractionated by electrophoresis at 110 V in 0.9 M Tris-borate and 2 mM EDTA, pH 8.0. Gels were dried and protein–DNA complexes were visualised by autoradiography and scanned on a Betagen Betascope 603 Blot analyser imaging system for quantitation of the retarded bands.

### *Sucrose density gradient analysis*

Nuclear extracts were isolated from Hs578T cells after incubation of a cell suspension with 10 nM [$^3$H]TCDD or 10 nM [$^3$H]TCDD plus a 200-fold excess of TCDF as described (Wang *et al*., 1992) and layered on linear sucrose gradients (5–25%) prepared in HEGD plus 0.4 M potassium chloride. Gradients were centrifuged at 404 000 *g* at 3°C for 2.5 h. After the centrifugation 30 fractions were collected from each gradient and radioactivity in each fraction was determined to give the total binding.

### *Statistical analysis*

Results are expressed as means±s.d. for at least three separate determinations for each experiment. Statistical significance was determined by ANOVA and Student's *t*-test and the levels of probability are noted.

### Results

After treatment of a suspension of Hs578T cells with 10 nM [$^3$H]TCDD for 2 h the nuclear extract was analysed by sucrose density gradient centrifugation. The results summarised in Figure 1 indicate that TCDD induces formation of a specifically bound nuclear AhR complex that is similar to that observed in other cell lines (Pasanen *et al*., 1988; Harris *et al*., 1989; Vickers *et al*., 1989; Thomsen *et al*., 1991). The results illustrated in Figure 2 show that nuclear extracts from untreated (DMSO) cells do not form an AhR–DRE complex with retarded mobility as determined in a gel electrophoretic mobility shift assay. After treatment of the cells with 10 nM TCDD, nuclear extracts formed a specific DNA–protein band with retarded mobility, which was decreased in intensity after incubation with 100-fold excess unlabelled DRE but was essentially unchanged by co-incubation with a 100-fold excess of unlabelled mutant DRE. Thus, the nuclear AhR that forms in Hs578T cells after treatment with TCDD (Figure 1) forms a complex with [$^{32}$P]DRE that can be detected using a gel retardation assay (Figure 2).

The effects of hER expression on restoration of Ah responsiveness in Hs578T cells was investigated in cells co-transfected with pRNH11c±hER. The results (Figure 3, Table I) indicate that in cells transfected with pRNH11c alone (Figure 3, lane 1), TCDD treatment resulted in only a

**Figure 1** Velocity sedimentation analysis of the nuclear TCDD–AhR complex in Hs578T cells. Cells in suspension were treated with 10 nM [$^3$H]TCDD ± 2 μM TCDF for 2 h; the cells were then collected, and nuclei were isolated, extracted and analysed by velocity sedimentation analysis as described in Materials and methods. Radiolabelled nuclear AhR complex sedimented at 6.9 S. ■, TCDD; ○, TCDD + TCDF.

**Figure 2** Gel mobility shift analysis of the AhR-DRE complex from Hs578T human breast cancer cells. The cells were treated with DMSO or 10 nM TCDD for 2 h at 37°C and analysed by gel electrophoretic mobility shift assay. Nuclear extracts from cells treated with DMSO (lane 1), TCDD (lane 2), TCDD plus competition with 100-fold excess of unlabelled DRE (lane 3) or mutant DRE (lane 4) were used in this assay. The relative band intensities in the specifically bound band (AhR–DRE complex) were lane 1, 5.1 ± 1.0; lane 2, 51.7 ± 3.4; lane 3, 3.3 ± 1.3; lane 4, 53.9 ± 4.0.

1.6-fold induction of CAT activity (Figure 3, lane 2). In cells co-transfected with the hER plasmid and pRNH11c, CAT activity in control (DMSO) cells was elevated 13.5-fold (Figure 3, lane 11) compared with transfection in the absence of hER. Co-transfection of hER and pRNH11c coupled with treatment with 10 nM TCDD also resulted in significantly increased CAT activity (Figure 3, lane 12); however, CAT activity was induced < 1.6-fold by TCDD. The possible restoration of Ah responsiveness in Hs578T cells was also investigated by co-transfecting cells with Arnt, AhR, Arnt plus AhR, Arnt plus AhR plus hER expression plasmids, and pRNH11c (Table I, Figure 3). Co-transfection of pRNH11c with Arnt, AhR or Arnt plus AhR expression plasmids resulted in no significant changes in basal CAT activity and minimal induction by TCDD. Increased basal (but not induced) CAT activity was only observed in cells transfected with hER. A similar set of experiments was carried out using pMCAT5.12, an Ah-responsive plasmid that contains DRE2 from the murine CYP1A1 gene (Table II). TCDD did not induce CAT activity in Hs578T cells transfected with pMCAT5.12. Co-transfection of cells with pMCAT5.12 and Arnt plus AhR expression plasmids did not affect basal or inducible (TCDD) CAT activity. However, in cells treated with DMSO and co-transfected with pMCAT5.12 with the hER, hER plus Arnt, hER plus AhR or hER plus AhR plus Arnt expression plasmids resulted in a 4- to 5-fold increase in CAT activity. CAT activity was not significantly induced by TCDD in the co-transfected Hs578T cells.

pRNH21c is derived from pRNH11c; however, the −831 to −560 nucleotides containing the NRE sequence have been deleted (Hines *et al.*, 1988). A comparison of the effects of hER on CAT activity in Hs578T cells co-transfected with pRNH11c or pRNH21c was also determined (Figure 4). The results obtained with pRNH11c were similar to those reported in Table I (Figure 4, lanes 1, 2, 5 and 6). In contrast, CAT activity in Hs578T cells co-transfected with pRNH21c ± hER was not detected in control (DMSO) cells or after treatment with TCDD.

The effects of hER and mutant hER plasmids with C-terminal deletions of amino acids 282 to 595 (HE15) or N-terminal deletions of amino acids 1–178 (HE19) on CAT activity in Hs578T cells transfected with pRNH11c were also investigated. The results (Table III) showed that in control (DMSO) cells co-transfected with pRNH11c plus HE15 or HE19 plasmids, there was a > 2- and > 47-fold decrease in CAT activity respectively, compared with cells co-transfected with pRNH11c plus hER. In Hs578T cells co-transfected with pRNH11c plus hER, HE15 or HE19 the effects of TCDD were dependent on the expressed ER or ER fragment. The results obtained using hER (full length) or HE15 were similar and CAT activity induced by TCDD was < 1.5-fold whereas a > 23-fold induction response was observed with HE19.

### Discussion

Studies in this laboratory have focused on determining the regulation of Ah responsiveness in human breast cancer cell lines using induction of CYP1A1 and inhibition of oestrogen-induced gene expression as models (Harris *et al.*, 1989; Arellano *et al.*, 1993; Moore *et al.*, 1993; Wang *et al.*, 1993; Thomsen *et al.*, 1994; Chaloupka *et al.*, 1995). Several reports suggest that induction of CYP1A1 in human breast cancer cells by AhR agonists requires a functional ER (Jaiswal *et al.*, 1985; Ivy *et al.*, 1988; Pasanen *et al.*, 1988; Harris *et al.*, 1989; Thomsen *et al.*, 1991, 1994). MDA-MB-231 cells are ER negative and Ah non-responsive; however, co-transfection of the hER plus the Ah-responsive pMCAT5.12 or pRNH11c plasmids resulted in a significant induction of CAT activity by TCDD (Thomsen *et al.*, 1994). Moreover, in a series of experiments that decrease transiently expressed ER, there was a corresponding decrease in Ah responsiveness. Hs578T cells have previously been characterised as ER negative and TCDD does not induce CYP1A1-dependent

**Figure 3** Effects of human AhR, Arnt and ER on restoration of Ah responsiveness in Hs578T human breast cancer cells. The cells were transiently transfected with 5 μg of pRNH11c (lanes 1 to 12) and co-transfected with 5 μg of other expression plasmids for each different experiment. Lanes 1, 3, 5, 7, 9 and 11 were derived from cells treated with DMSO, whereas lanes 2, 4, 6, 8, 10 and 12 were treated with 10 nM TCDD for 44 h. The various treatments are indicated in the Figure 3 and quantitation of induced CAT activities are summarised in Table I.

**Table I** Comparative effects of human AhR, Arnt and hER expression plasmids on restoring Ah responsiveness to THs578Tcells by transient co-transfection studies with the pRNH11c plasmid[a]

| | *Relative CAT activity* | |
|---|---|---|
| *Transfected plasmids* | *DMSO* | *TCDD* |
| pRNH11c | 1 | 1.6±0.2 |
| pRNH11c+Arnt | 1.9±0.2 | 2.7±0.3 |
| pRNH11c+AhR | 1.0±0.1 | 3.0±0.4 |
| pRNH11c+Arnt+AhR | 1.5±0.3 | 2.2±0.5 |
| pRNH11c+Arnt+AhR+hER | 15.6±1.7[b] | 19.6±0.9[b] |
| pRNH11c+hER | 13.5±1.2[b] | 20.5±0.8[b] |

[a] The cells were transfected with 5 μg of each plasmid for each individual group, shocked with 25% DMSO and dosed with DMSO or 10 nM TCDD for 44 h and standardised against DMSO-treated Hs578T cells that were transfected with pRNH11c plasmid alone. [b] Statistically higher ($P<0.01$) than DMSO-treated Hs578T cells transfected with pRNH11c.

**Table II** Comparative effects of human AhR, Arnt and hER expression plasmids on restoring Ah responsiveness to Hs578T cells by transient co-transfection studies with the pMCAT5.12 plasmid[a]

| | *Relative CAT activity* | |
|---|---|---|
| *Transfected plasmids* | *DMSO* | *TCDD* |
| pMCAT5.12 | 1 | 1.1±0.3 |
| pMCAT5.12+hER | 5.4+0.5[b] | 6.7+0.6[b] |
| pMCAT5.12+hER+Arnt | 4.1+0.6[b] | 5.4±0.5[b] |
| pMCAT5.12+hER+AhR | 5.0±0.3[b] | 5.6±0.3[b] |
| pMCAT5.12+Arnt+AhR | 1.3+0.2 | 1.4±0.3 |
| pMCAT5.12+Arnt+AhR+hER | 4.3+0.7[b] | 6.1+0.6[b] |

[a] The cells were transfected with 5 μg of each plasmid for each individual group, shocked with 25% DMSO and dosed with DMSO or 10 nM TCDD for 44 h and standardised against DMSO-treated Hs578T cells that were transfected with pMCAT5.12 plasmid alone. [b] Statistically higher ($P<0.01$) than DMSO-treated Hs578T cells transfected with pMCAT5.12.

activity in this cell line (Arellano *et al.*, 1993). However, nuclear extracts from cells treated with [3H]TCDD or unlabelled TCDD form a specifically bound 6.9 S nuclear AhR complex (Figure 1) that binds to a [32P]DRE to give a band with retarded mobility in a gel electrophoretic mobility shift assay (Figure 2). These results suggest that the Ah non-responsiveness of Hs578T cells is not due to the failure of these cells to express the AhR and form a nuclear 6.9 S AhR complex or to interact with DREs. The results are in contrast to mutant benzo[*a*]pyrene-resistant MCF-7 breast cancer cells that also express the nuclear AhR but do not bind to a DRE (Moore *et al.*, 1993). The failure of the AhR to form a DRE complex is consistent with the Ah non-responsiveness of the mutant MCF-7 cells; however, the results obtained with Hs578T cells indicate that other factors must be associated with the failure to observe an induction response with TCDD. Since cycloheximide treatment of Hs578T cells also does not restore induction of CYP1A1 mRNA levels by TCDD (Arellano *et al.*, 1993), it is unlikely that a labile protein or related factor is involved in the repressed induction response.

The effects of ER expression on Ah responsiveness in Hs578T cells were investigated by co-transfecting an hER expression plasmid with pRNH11c that contains the −1142 to +2434 sequence from the human CYP1A1 gene fused to a bacterial CAT reporter gene (Hines *et al.*, 1988). The results (Figure 3 and Table I) show that transient ER expression significantly increases basal CAT activity in untreated (DMSO) cells; however, induction of CAT activity by TCDD was minimal. In a series of transient transfection studies using expression plasmids for Arnt, AhR and ER or their combinations, the major response was a significant increase in basal CAT activity only in the presence of hER; in contrast, minimal induction of CAT activity by TCDD was observed. Similar results were obtained using pMCAT5.12, an Ah-responsive plasmid that contains the murine DRE2 but not the extensive 5′-regulatory DNA fragment associated with the pRNH11c plasmid. These data are in contrast to previous transient transfection studies with MDA-MB-231 cells in which transient expression of ER restored Ah responsiveness with both pRNH11c and pMCAT5.12 plasmids but did not affect basal CAT activity (Thomsen *et al.*, 1994). Preliminary studies with MDA-MB-231 cells also show that the Arnt expression plasmid also partially restores Ah responsiveness (unpublished results) whereas this was not observed in Hs578T cells co-transfected with the Arnt expression plasmid (Tables I and II).

An NRE has been identified in the 5′-promoter of the human CYP1A1 gene (−833 to −558) (Hines *et al.*, 1988; Boucher *et al.*, 1993) and the pRNH21c construct has the NRE sequence deleted. In transient transfection studies with Hep G2 human hepatoma cells with both pRNH11c and

**Figure 4** Effects of hER expression on restoration of Ah responsiveness by co-transfection with pRNH11c or pRNH21C. Aliquots of 5 $\mu$g of each plasmid were used for transient transfection. Lanes 1, 3, 5 and 7 were treated with DMSO, whereas lanes 2, 4, 6 and 8 were treated with 10 nM TCDD for 44 h. Relative CAT activities observed in cells transiently transfected with pRNH11c + hER and pRNH21c + hER were $1.0 \pm 0.2$ (DMSO) and $1.1 \pm 0.1$ (TCDD) and $0.04 \pm 0.01$ (DMSO) and $0.04 \pm 0.005$ (TCDD) respectively. CAT activities in cells transfected with pRNH21c alone were not significantly different than results obtained in cells co-transfected with pRNH21c + hER.

**Table III** Comparative effects of human wild- and mutant-type ER expression plasmids on restoring Ah responsiveness to Hs578Tcells by transient co-transfection studies with Ah-responsive pRNH11c plasmid[a]

| *Transfected plasmids* | *Relative CAT activity* *DMSO* | *TCDD* |
|---|---|---|
| pRNH11c + hER | 100 | 151.9[b] |
| pRNH11c + HE15 | 45.9[b] | 51.9[b] |
| pRNH11c + HE19 | 2.1[b] | 48.9[b] |

[a] The cells were transfected with 5 $\mu$g of each plasmid for each individual group, shocked with 25% DMSO and dosed with DMSO or 10 nM TCDD for 44 h and standardised against DMSO-treated Hs578T cells that were co-transfected with pRNH11c plasmid alone and the full-length or truncated ER expression plasmids. [b] Statistically higher ($P < 0.01$) than DMSO-treated Hs578T cells co-transfected with pRNH11c plus ER plasmids.

pRNH21c, there was an 8.4-fold increase in basal CAT activity after deletion of the NRE and this was accompanied by a significant decrease in the fold induction of CAT activity by polynuclear aromatic hydrocarbons using pRNH21c (Hines *et al*., 1988). Based on these results from Hep G2 cells, it was hypothesised that in Hs578T cells, ER expression may derepress the effects of the NRE on the basal activity of the CYP1A1 promoter. However, a comparison of basal and induced CAT activity in Hs578T cells co-transfected with hER plus pRNH11c or pRNH21c indicates that CAT activity was minimal using the NRE-deleted pRNH21c in the presence or absence of TCDD (Figure 4). These results illustrate differences in the role of the NRE and/or NRE-associated proteins in promoter-dependent regulation of CYP1A1 in Hs578T human breast cancer and Hep G2 human hepatoma cell lines.

The ER contains several structural domains, including at least two important transactivation regions, TAF1 and TAF2, that are associated with constitutive and ligand-inducible activities respectively (Kumar *et al*., 1987). Previous studies with MDA-MB-231 cells co-transfected with pRNH11c plus hER, HE15 or HE19 showed that Ah responsiveness was restored by expression of either the full length or both truncated ERs (Thomsen *et al*., 1994). The results summarised in Table III demonstrate that using the same experimental design with Hs578T cells gave results that were in contrast to those reported for MDA-MB-231 cells. Expression of C-terminal-deleted ER (HE15) in Hs578T cells increased basal CAT activity but not Ah responsiveness, whereas expression of the N-terminal-deleted ER (HE19) resulted in a >47-fold loss of basal activity but restoration of Ah responsiveness, since TCDD caused a 23-fold increase in CAT activity. These results suggest that in Hs578T cells, the various domains of the ER play a differential role in restoration of Ah responsiveness. The predominant effect of the ER and the C-terminal-deleted ER is to increase basal but not inducible activity regulated by the CYP1A1 promoter in pRNH11c. However, expression of N-terminal-deleted ER (HE19) resulted in a dramatic loss of basal CAT activity but restoration of Ah responsiveness in Hs578T cells co-transfected with pRNH11c plus HE19 (Table III). Thus, expression of amino acids 179 to 595 of the ER is sufficient to restore Ah responsiveness to Hs578T cells and eliminate the overriding ER-mediated increase in basal activity, which appears to be primarily associated with the N-terminal portion of the ER. Previous studies have reported higher basal or constitutive expression of CYP1A1 in some breast tumours and this elevated response may be useful as a negative prognostic indicator for breast cancer (Murray *et al*., 1991; Pyykkö *et al*., 1991). The results observed in this study with Hs578T cells demonstrate that expression of the full-length or C-terminal-deleted ER significantly increases constitutive CYP1A1 activity. It has recently been reported that exon 5 deletion variant ER (△5ER) mRNA is overexpressed in some tumours and the resulting protein contains TAF-1 but lacks TAF-2 and the ligand-binding domain of the ER (Fuqua *et al*., 1993; Daffada *et al*., 1995; Villa *et al*., 1995). These observations are consistent with the enhancement of basal CYP1A1-dependent activity in Hs578T cells by HE15, which is functionally similar to △5ER and suggests that future studies on the linkage between expression of △5ER and high basal CYP1A1 activity in breast tumours is warranted.

In summary, the results of this study with ER-negative Hs578T cells illustrate that regulation of CYP1A1 is highly variable in human breast cancer cell lines. The restoration of Ah responsiveness in Hs578T cells by truncated ER-encoding amino acids 179 to 595 suggests that the ligand-dependent TAF-2 (Kumar *et al.*, 1987) may play an important role in this response. Current studies in this laboratory are focused on delineating the cell-specific regulation of CYP1A1 in ER-positive and ER-negative human breast cancer cells and delineating the protein–protein and protein–DNA interactions that are required for transactivation of CYP1A1.

**Acknowledgements**
The financial assistance of the National Institutes of Health (ES03843) and the Texas Agricultural Experiment Station is gratefully acknowledged. S Safe is a Sid Kyle Professor of Toxicology.

### References

ANTTILA S, HIETANEN E, VAINIO H, CAMUS A, GELBOIN HV, PARK SS, HEIKKILA L, KARJALAINEN A AND BARTSCH H. (1991). Smoking and peripheral type of cancer are related to high levels of pulmonary cytochrome P4501A in lung cancer patients. *Int. J. Cancer*, **47**, 681–685.

ARELLANO LO, WANG X AND SAFE S. (1993). Effects of cycloheximide on the induction of *CYP1A1* gene expression by 2,3,7,8-tetrachlorodibenzo-*p*-dioxin (TCDD) in three human breast cancer cell lines. *Carcinogenesis*, **14**, 219–222.

BOUCHER PD, RUCH RJ AND HINES RN. (1993). Specific nuclear protein binding to a negative regulatory element on the human *CYP*1A1 gene. *J. Biol. Chem.*, **268**, 17384–17391.

BRADFORD MM. (1976). A rapid and sensitive method for the quantitation of microgram quantities of protein utilizing the principle of protein-dye binding. *Anal. Biochem.*, **72**, 248–254.

BURBACH KM, POLAND AB AND BRADFIELD CA. (1992). Cloning of the Ah-receptor cDNA reveals a distinctive ligand-activated transcription factor. *Proc. Natl Acad. Sci. USA*, **89**, 8185–8189.

CHALOUPKA K, STEINBERG M, SANTOSTEFANO M, RODRIGUEZ LV, GOLDSTEIN L AND SAFE S. (1995). Induction of Cyp1a-1 and Cyp1a-2 gene expression by a reconstituted mixture of polynuclear aromatic hydrocarbons in B6C3F1 mice. *Chem.-Biol., Interact.*, **96**, 207–221.

DAFFADA AAI, JOHNSTON SRD, SMITH IE, DETRE S, KING N AND DOWSETT M. (1995). Exon 5 deletion variant estrogen receptor messenger RNA expression in relation to tamoxifen resistance and progesterone receptor/pS2 status in human breast cancer. *Cancer Res.*, **55**, 288–293.

DENISON MS AND DEAL RM. (1990). The binding of transformed aromatic hydrocarbon (Ah) receptor to its DNA recognition site is not affected by metal depletion. *Mol. Cell Endocrinol.*, **69**, 51–57.

EMA M, SOGAWA K, WATANABE N, CHUJOH Y, MATSUSHITA N, GOTOH O, FUNAE Y AND FUJII-KURIYAMA Y. (1992). cDNA cloning and structure of the putative Ah receptor. *Biochem. Biophys. Res. Comm.*, **184**, 246–253.

FOLDES RL AND BRESNICK E. (1989). Inducibility of rat liver cytochrome P-450IA1 (P-450c) mRNA during the partial inhibition of protein synthesis. *Biochem. Pharmacol.*, **38**, 1017–1019.

FUJISAWA-SEHARA A, SOGAWA K, YAMANE M AND FUJII-KURIYAMA Y. (1987). Characterization of xenobiotic responsive elements upstream from the drug-metabolizing cytochrome P-450c gene: a similarity to glucocorticoid regulatory elements. *Nucleic Acids Res.*, **15**, 4179–4191.

FUQUA SAW, ALLRED DC AND AUCHUS RJ. (1993). Expression of estrogen receptor variants. *J. Cell. Biochem.*, **17G**, 194–197.

GONZALEZ FJ AND NEBERT DW. (1985). Autoregulation plus upstream positive and negative control regions associated with transcriptional activation of the mouse cytochrome $P_1$-450 gene. *Nucleic Acids Res.*, **13**, 7269–7288.

GRADIN K, WILHELMSSON A, POELLINGER L AND BERGHARD A. (1993). Nonresponsiveness of normal fibroblasts to dioxin correlates with the presence of a constitutive xenobiotic response element-binding factor. *J. Biol. Chem.*, **268**, 4061–4068.

HARRIS M, PISKORSKA-PLISZCZYNSKA J, ZACHAREWSKI T, ROMKES M AND SAFE S. (1989). Structure-dependent induction of aryl hydrocarbon hydroxylase in human breast cancer cell lines and characterization of the Ah receptor. *Cancer Res.*, **49**, 4531–4535.

HINES RN, MATHIS JM AND JACOB CS. (1988). Identification of multiple regulatory elements on the human cytochrome P4501A1 gene. *Carcinogenesis*, **9**, 1599–1605.

HOFFMAN EC, REYES H, CHU F, SANDER F, CONLEY LH, BROOKS BA AND HANKINSON O. (1991). Cloning of a factor required for activity of the Ah (dioxin) receptor. *Science*, **252**, 954–958.

IVY SP, TULPULE A, FAIRCHILD CR, AVERBUCH SD, MYERS CE, NEBERT DW, BAIRD WM AND COWAN KH. (1988). Altered regulation of P-4501A1 expression in a multidrug-resistant MCF-7 human breast cancer cell line. *J. Biol. Chem.*, **263**, 19119–19125.

JAISWAL AK, GONZALEZ FJ AND NEBERT DW. (1985). Human dioxin-inducible cytochrome $P_1$-450: complementary DNA and amino acid sequence. *Science*, **228**, 80–83.

JONES PB, GALEAZZI DR, FISHER JM AND WHITLOCK JP, JR. (1985). Control of cytochrome $P_1$-450 gene expression by dioxin. *Science*, **227**, 1499–1502.

JONES PB, DURRIN LK, GALEAZZI DR AND WHITLOCK JP, JR. (1986). Control of cytochrome $p_1$-450 gene expression: analysis of a dioxin-responsive enhancer system. *Proc. Natl Acad. Sci. USA*, **83**, 2802–2806.

KAWAI S AND NISHIZAWA M. (1984). New procedure for DNA transfection with polycation and dimethyl sulfoxide. *Mol. Cell Biol.*, **4**, 1172–1174.

KAWAJIRI K AND FUJIIKURIYAMA Y. (1991). P450 and human cancer. *Jpn. J. Cancer Res.*, **82**, 1325–1335.

KELLERMANN G, SHAW CR AND LUYTEN-KELLERMANN M. (1973). Aryl hydrocarbon hydroxylase inducibility and bronchogenic carcinoma. *N. Engl. J. Med.*, **189**, 934–937.

KELSEY KT, WIENCKE JK AND SPITZ MR. (1994). A race-specific genetic polymorphism in the CYP1A1 gene is not associated with lung cancer in African Americans. *Carcinogenesis*, **15**, 1121–1124.

KUMAR V, GREEN S, STACK G, BERRY M, JIN J AND CHAMBON P. (1987). Functional domains of the human estrogen receptor. *Cell*, **51**, 941–951.

LUSSKA A, WU L AND WHITLOCK JP, JR. (1992). Superinduction of *CYP1A1* transcription by cycloheximide: role of the DNA binding site for the liganded Ah receptor. *J. Biol. Chem.*, **267**, 15146–15151.

MANIATIS T, FRITSCH EF AND SAMBROOK J. (1982). *Molecular Cloning: A Laboratory Manual*. pp. 187–210. Cold Spring Harbor Press: Cold Spring Harbor, NY.

MOORE M, NARASIMHAN TR, STEINBERG M, WANG X AND SAFE S. (1993). Potentiation of *CYP1A1* gene expression in MCF-7 human breast cancer cells cotreated with 2,3,7,8-tetrachlorodibenzo-*p*-dioxin and 12-O-tetradecanoylphorbol-13-acetate. *Arch. Biochem. Biophys.*, **305**, 483–488.

MORGAN TL, MAHER VM AND MCCORMICK JJ. (1986). Optimal parameters for the polybrene-induced DNA transfection of diploid human fibroblasts. *In Vitro Cell Dev. Biol.*, **22**, 317–319.

MURRAY GI, FOSTER CO, BARNES TS, WEAVER RJ, EWEN SWB, MELVIN WT AND BURKE MD. (1991). Expression of cytochrome P4501A in breast cancer. *Br. J. Cancer*, **63**, 1021–1023.

NAKACHI K, IMAI K, HAYASHI S AND KAWAJIRI K. (1993). Polymorphisms of the *CYP1A1* and glutathione S-transferase genes associated with susceptibility to lung cancer in relation to cigarette dose in a Japanese population. *Cancer Res.*, **53**, 2994–2999.

NELSON DR, KAMATAKI T, WAXMAN DJ, GUENGERICH FP, ESTABROOK RW, FEYEREISEN R, GONZALEZ FJ, COON MJ, GUNSALUS IC, GOTOH O, OKUDA K AND NEBERT DW. (1993). The P450 Superfamily: update on new sequences, gene mapping, accession numbers, early trivial names of enzymes and nomenclature. *DNA Cell Biol.*, **12**, 1–51.

NEMOTO N AND SAKURAI J. (1991). Increase of CYP1A1 mRNA and AHH activity by inhibitors of either protein or RNA synthesis in mouse hepatocytes in primary culture. *Carcinogenesis*, **12**, 2115–2121.

PASANEN M, STACEY S, LYKKESFELDT A, BRIAND P, HINES R AND AUTRUP H. (1988). Induction of cytochrome P-4501A1 gene expression in human breast tumor cells. *Chem.-Biol. Interact.*, **66**, 223–232.

PYYKKÖ K, TUIMALA R, AALTO L AND PERKIÖ T. (1991). Is aryl hydrocarbon hydroxylase activity a new prognostic indicator for breast cancer. *Br. J. Cancer*, **63**, 596-600.

REICK M, ROBERTSON RW, PASCO DS AND FAGAN JB. (1994). Down-regulation of nuclear aryl hydrocarbon receptor DNA-binding and transactivation functions: requirement for a labile or inducible factor. *Mol. Cell Biol.*, **14**, 5653-5660.

REYES H, REISZ-PORSZASZ S AND HANKINSON O. (1992). Identification of the Ah receptor nuclear translocator protein (Arnt) as a component of the DNA binding form of the Ah receptor. *Science*, **256**, 1193-1195.

ROBERTSON RW, ZHANG L, PASCO DS AND FAGAN JB. (1994). Aryl hydrocarbon-induced interactions at multiple DNA elements of diverse sequence-a multicomponent mechanism for activation of cytochrome P4501A1 (CYP1A1) gene transcription. *Nucleic Acids Res.*, **22**, 1741-1749.

SIVARAMAN L, LEATHAM MP, YEE J, WILKENS LR, LAU AF AND LE MARCHAND L. (1994). *CYP1A1* genetic polymorphisms and *in situ* colorectal cancer. *Cancer Res.*, **54**, 3692-3695.

STERLING K, WEAVER J, HO KL, XU LC AND BRESNICK E. (1993). Rat *CYP1A1* negative regulatory element: biological activity and interaction with a protein from liver and hepatoma cells. *Mol. Pharmacol.*, **44**,560-568.

SWANSON HI AND BRADFIELD CA. (1993). The Ah-receptor: genetics, structure and function. *Pharmacogenetics*, **3**, 213-223.

TAIOLI E, CROFTS F, TRACHMAN J, DEMOPOULOS R, TONIOLO P AND GARTE SJ. (1995). A specific African-American *CYP1A1* polymorphism is associated with adenocarcinoma of the lung. *Cancer Res.*, **55**, 472-473.

THOMSEN JS, NISSEN L, STACEY SN, HINES RN AND AUTRUP H. (1991). Differences in 2,3,7,8-tetrachlorodibenzo-*p*-dioxin-inducible CYP1A1 expression in human breast carcinoma cell lines involve altered transacting factors. *Eur. J. Biochem.*, **197**, 577-582.

THOMSEN JS, WANG X, HINES RN AND SAFE S. (1994). Restoration of Ah responsiveness in MDA-MB-231 human breast cancer cells by transient expression of the estrogen receptor. *Carcinogenesis*, **15**, 933-937.

VICKERS PJ, DUFRESNE MJ AND COWAN KH. (1989). Relation between cytochrome P4501A1 expression and estrogen receptor content of human breast cancer cells. *Mol. Endocrinol.*, **3**, 157-164.

VILLA E, CAMELLINI L, DUGANI A, ZUCCHI F, GROTTOLA A, MERIGHI A, BUTTAFOCO P, LOSI L AND MANENTI F. (1995). Variant estrogen receptor messenger RNA species detected in human primary hepatocellular carcinoma. *Cancer Res.*, **55**, 498-500.

WANG X, ROSENGREN R, MORRISON V AND SAFE S. (1992). Characterization of the aryl hydrocarbon receptor in the human C-4II cervical squamous carcinoma cell line. *Biochem. Pharmacol.*, **43**, 1635-1642.

WANG X, PORTER W, KRISHNAN V, NARASIMHAN TR AND SAFE S. (1993). Mechanism of 2,3,7,8-tetrachlorodibenzo-*p*-dioxin (TCDD)-mediated decrease of the nuclear estrogen receptor in MCF-7 human breast cancer cells. *Mol. Cell. Endocrinol.*, **96**, 159-166.

WATSON AJ, WEIR-BROWN KI, BANNISTER RM, CHU F, REISZ-PORSZASZ S, FUJII-KURIYAMA Y, SOGAWA K AND HANKINSON O. (1992). Mechanism of action of a repressor of dioxin-dependent induction of *Cyp1a1* gene transcription. *Mol. Cell Biol.*, **12**, 2115-2123.

WHITELAW M, PONGRATZ I, WILHELMSSON A, GUSTAFSSON JA AND POELLINGER L. (1993). Ligand-dependent recruitment of the arnt coregulator determines DNA recognition by the dioxin receptor. *Mol. Cell Biol.*, **13**, 2504-2514.

WHITLOCK JP, JR. (1993). Mechanistic aspects of dioxin action. *Chem. Res. Toxicol.*, **6**, 754-763.